# Prognostic value and multifaceted roles of tetraspanin CD9 in cancer

**DOI:** 10.3389/fonc.2023.1140738

**Published:** 2023-03-17

**Authors:** Róbert Ondruššek, Barbora Kvokačková, Karolína Kryštofová, Světlana Brychtová, Karel Souček, Jan Bouchal

**Affiliations:** ^1^ Department of Clinical and Molecular Pathology, Institute of Molecular and Translational Medicine, Faculty of Medicine and Dentistry, Palacky University, Olomouc, Czechia; ^2^ Department of Pathology, EUC Laboratore CGB a.s., Ostrava, Czechia; ^3^ Department of Cytokinetics, Institute of Biophysics of the Czech Academy of Sciences, Brno, Czechia; ^4^ International Clinical Research Center, St. Anne’s University Hospital, Brno, Czechia; ^5^ Department of Experimental Biology, Faculty of Science, Masaryk University, Brno, Czechia; ^6^ Proteomics Core Facility Central European Institute of Technology, Masaryk University, Brno, Czechia; ^7^ National Centre for Biomolecular Research, Faculty of Science, Masaryk University, Brno, Czechia; ^8^ Department of Clinical and Molecular Pathology, University Hospital Olomouc, Olomouc, Czechia

**Keywords:** CD9, cancer, immunohistochemistry, prognosis, exosomes

## Abstract

CD9 is a crucial regulator of cell adhesion in the immune system and plays important physiological roles in hematopoiesis, blood coagulation or viral and bacterial infections. It is involved in the transendothelial migration of leukocytes which might also be hijacked by cancer cells during their invasion and metastasis. CD9 is found at the cell surface and the membrane of exosomes affecting cancer progression and therapy resistance. High expression of CD9 is mostly associated with good patients outcome, with a few exceptions. Discordant findings have been reported for breast, ovarian, melanoma, pancreatic and esophageal cancer, which might be related to using different antibodies or inherent cancer heterogeneity. According to *in vitro* and *in vivo* studies, tetraspanin CD9 is not clearly associated with either tumor suppression or promotion. Further mechanistic experiments will elucidate the role of CD9 in particular cancer types and specific conditions.

## 1 Introduction

**Figure 1:**
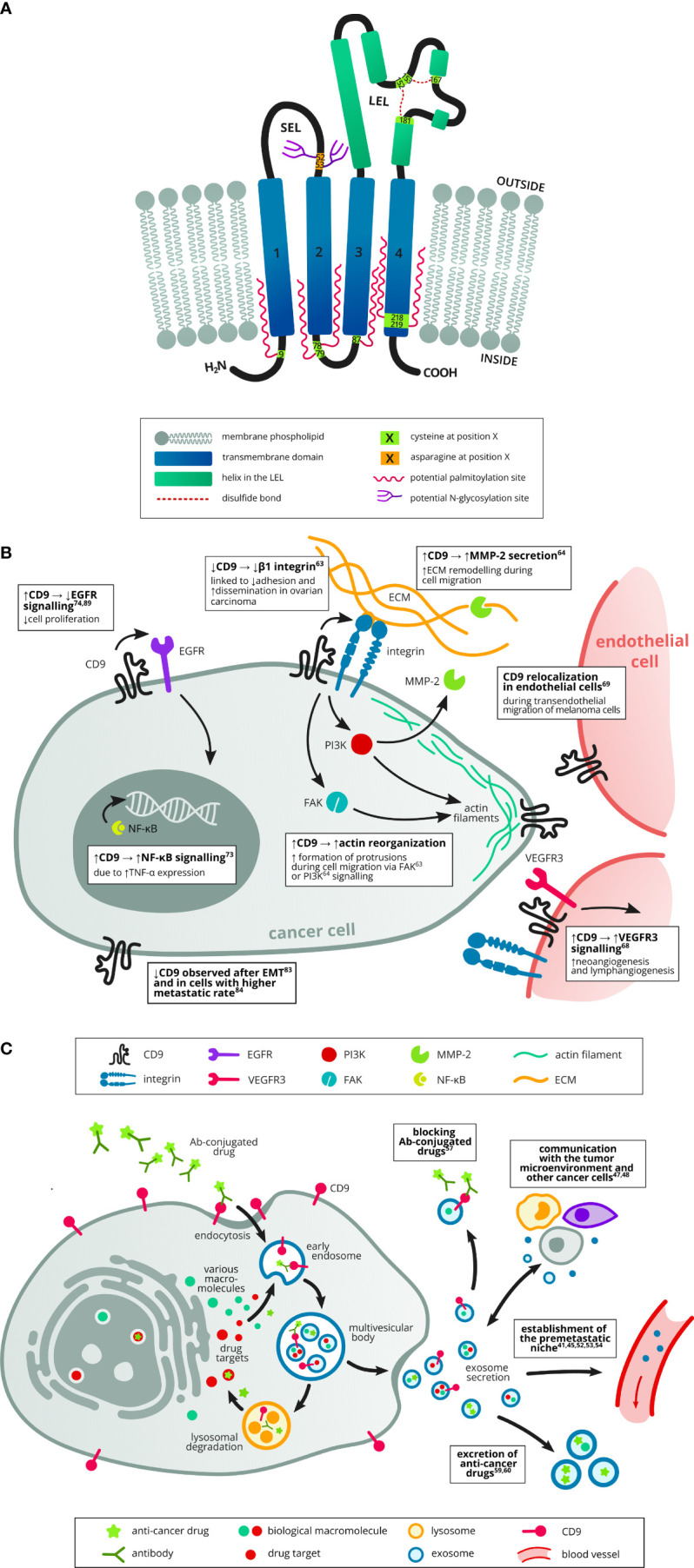
Structure of tetraspanin CD9 and its role in cancer, including exosome trafficking. **(A)** The CD9 protein consists of four transmembrane domains (1–4), short (EC1, SEL) and long (EC2, LEL) extracellular loop, short intracellular loop and short intracellular N- and C-termini. There are several possible palmitoylation sites made up of membrane-proximal cysteines and a possible N-glycosylation site in the SEL. In the LEL, there are two disulfide bridges, each containing one cysteine of the CCG motif (152–154), a typical feature of the tetraspanin family. Based on UniProt (AC: P21926, cited 1.8.2022). **(B)** CD9 was implicated both in tumor promoting and suppressing mechanisms. Several studies have described its role in cell migration and invasion, e.g. by affecting actin-polymerization and reorganisation at the cell protrusions (5, 6) or by increasing the production of the proteinase MMP-2 which cleaves ECM components during cell invasion (6). Increased CD9 expression was also linked to increased signalling in the protumorigenic NF-κB pathway (7). However, increased CD9 expression was shown to attenuate EGFR signalling and thus suppress cell proliferation (8, 9). Another study described higher metastatic rate in cells with decreased CD9 expression (10). CD9 downregulation was also observed in cells which underwent EMT (11). CD9 can also affect tumor neoangiogenesis by promoting VEGFR3 signalling in endothelial cells (12). Last but not least, transendothelial migration of tumor cells is supported by CD9 reorganisation at points of contact between endothelial and tumor cells (13). **(C)** Exosomes can transport cargo between cells in the tumor microenvironment (other tumor cells, stromal cells, immune cells) and thus enable mutual communication (14, 15). They also help establish the premetastatic niche in the target organ before colonization (16–20). Exosomes can also promote drug resistance *via* several mechanisms, e.g. by transporting drugs out of the tumor cells (21, 22) or by neutralisation of antibody-conjugated drugs (23).

Tetraspanin CD9, also known as TSPAN29 or motility-related protein 1, is a member of the transmembrane 4 superfamily proteins, which are characterized by four transmembrane domains, two extracellular loops, and short intracellular N-and C-terminal tails (**Figure 1A**) (1, 2). Like other tetraspanins, CD9 can undergo palmitoylation on each of its membrane-proximal cysteines which affects its interactions with other partners (3). Tetraspanins generally form tetraspanin-enriched microdomains (TEMs) in cell membranes. Within these domains, they interact with various transmembrane and intracellular partners, including other tetraspanins, integrins, proteases, immunoglobulins, and intracellular signaling proteins (4). Therefore, the biological effects of CD9 depend on these dynamic interactions within the context of TEMs (1, 24). Recently, a “concatenation model” for forming CD9/EWI-F assemblies has been suggested, which may explain the occurrence of these TEMs (25). Besides tetraspanins (e.g. CD63, CD81, CD151 and TSPAN4), CD9 interacts with numerous single-span transmembrane proteins, such as integrins (e.g. CD49c/ITGA3 and CD29 (1, 26), immunoglobulin superfamily proteins (e.g. EWI-F/PTGFRN and EWI-2/IGSF8) (1, 27), heparin-binding EGF-like growth factor (28), and metalloprotease ADAM17 (A Disintegrin And Metalloproteinase 17) (29). Previously, CD9 has also shown the ability to interact with other proteins such as CD19, CD46 and CD117 (30–32).

An equally important role is played by the interaction of tetraspanins with intracellular signaling molecules, although significantly fewer of them have been identified compared to transmembrane partners (4). In the context of CD9, these are mainly interactions with small GTPases of the Rho family (Rac and RhoA) that affect the actin cytoskeleton (33, 34), ERM proteins (ezrin-radixin-moesin) that mediate binding with the cytoskeleton, and PKC (35), which regulates the function of a wide range of proteins and intracellular signaling. Obviously, tetraspanin CD9 plays a complex role both in physiological conditions as well as in many diseases including cancer (**Figure 1B**).

## 2 Physiological roles of CD9

CD9 is a key regulator of cell adhesion in the immune system and plays an important role in the physiology of leukocytes and endothelial cells as well as in hematopoiesis and blood coagulation. Other physiological processes with the important role of CD9 include sperm-egg fusion (36), neurite outgrowth (37) or myotube formation (38). Recently, CD9 and tetraspanin 4 were revealed as membrane curvature sensors which play an essential role in the formation of migrasome and fertilization (39). In this sense, it was also shown that the reversed cone-like molecular shape of CD9 generates membrane curvature in the crystalline lipid layers, which explains the CD9 localization in regions with high membrane curvature and its implications in membrane remodeling (40). CD9 is also an exosomal marker and may affect the cellular exosomal transport and interactions between tumor cells and the stromal microenvironment (see chapter below).

One of the important functions of CD9 is the regulation of hematopoietic stem cell differentiation in the bone marrow and critical hematopoiesis events. CD9 is essential in megakaryocytic (41), lymphoid and myeloid differentiation (42). *In vitro* work proved that immature CD34+ bone marrow cells express high levels of CD9, while differentiated cells lose their expression (41). Also, CD9-expressing stromal cells of bone marrow affect hematopoietic cells and may be one factor that determines the degree of stem cell differentiation (43). Next, CD9 is involved in the blood clotting process, because it is a component of integral membrane proteins expressed on the cell surface and granular membranes of thrombocytes, which play an essential role in the coagulation process. It is part of an alpha 2b/beta 3 - CD9 - CD63 integrin - tetraspanin complex in activated platelets (44, 45).

The role of CD9 in different immune cells and its relevance to inflammation has recently been reviewed by Brosseau et al. (2). For example, the tetraspanin CD9 plays an important role in the immune synapse in two ways:/1/Through its association with LFA-1 on the T cell, CD9 controls the state of aggregation and adhesive capacity of this integrin./2/On the surface of the antigen-presenting cells, CD9 recruits ICAM-1 into TEMs, thus increasing its adhesive capacity (1). Its importance was also shown for the endothelial receptors such as integrin ligands ICAM-1 and VCAM-1, which facilitate leukocyte adhesion to the endothelium and their subsequent transmigration. Another important CD9 function is the inhibition of ADAM10 and ADAM17 sheddase activity, which enhances cell-cell adhesion and costimulatory capacity (1).

CD9 also affects viral (46) and bacterial infections (47, 48). Within TEMs, CD9 modulates various virus-induced processes at the membrane, including membrane fusion, viral budding and viral release. Sims et al. demonstrated that exosomes could enhance HIV-1 entry into human T and monocytic cell lines *via* exosomal tetraspanin proteins CD81 and CD9 (46). Infection by enveloped coronaviruses initiates with viral spike proteins binding to cellular receptors and is followed by proteolytic cleavage which prompts virus-cell membrane fusion. Infection, therefore, requires the proximity of receptors and proteases which ensures that virus-cell entry occurs at the appropriate time and place. Earnest et al. showed that CD9 is crucial for condensing these receptors and proteases (DPP4 and TMPRSS2, respectively) which allows viruses to enter cells efficiently and rapidly (49, 50). Although it might be reasonable to think that SARS-CoV-2 virulence relies on CD9 activity by clustering and scaffolding receptor and protease (i.e. ACE2 and TMPRSS2, respectively) for efficient cell entry, this hypothesis has not been validated yet in current literature (50, 51).

## 3 CD9 in exosomes and cell-to-cell communication

There is a growing interest in cell-cell communication mediated by secreted vesicles termed exosomes (**Figure 1C**) (52). Exosomes are nanoscaled extracellular vesicles (EVs) (generally, their sizes range from 30 to 150 nm) released by almost all cell types. Tetraspanins are an abundant component of exosome membranes, with CD9 being one of the most frequently found along with CD63, CD81, CD82 and TSPAN8 (53, 54). Functionally it has been demonstrated that CD9 knock-down in extracellular vesicles from breast cancer cells or recipient cells reduced endocytosis (55). Importantly, tetraspanins can influence the composition of exosomes through interactions with their binding partners. For example, a decrease in CD9 expression led to a significant reduction in the metalloprotease CD10 content in exosomes of pre-B-lymphocytes (56). CD10 can serve as a positive (57) and negative (58) prognostic factor in some cancers, but its role in exosomes has not yet been described. It has also been shown that the alteration of CD9 and CD151 on prostate cells alters the proteome of their resultant EVs and that these EVs can enhance the migratory and invasive capabilities of a non-tumorigenic prostate cellular population (59). The cargo of exosomes reflects the state of tumor cells from which they are derived. They can be explored as minimally invasive biomarkers for the early detection, diagnosis and prognosis of various cancers (16, 44, 60). Currently, there are many methods for exosome isolation and detection, ranging from classical ultracentrifugation or filtration to immunoaffinity, flow cytometry and acoustics-based microfluidic techniques (61, 62).

The mechanisms of exosome biogenesis are highly regulated through several distinct pathways, including ESCRT (endosomal sorting complexes required for transport) ‐ dependent and ESCRT‐independent pathways (17). Although the exosome release is a physiological process, its increased rate and specific cargo are favorable for oncogenic progression and metastases (16). Regarding CD9, its alterations affect extracellular vesicle secretion and mitophagy in melanoma cells (63). The exosome‐mediated communication is not limited to the cancer cells, it has also been shown in different cell types within the tumor microenvironment locally and distantly. Bioactive molecules, including CD9 in exosomes derived from cancer and stromal cells, provide the essential signals for the re-education of various cells and remodeling the tumor architecture (14, 15). For instance, in pancreatic ductal adenocarcinoma, CD9 mediated EV uptake from cancer-associated fibroblasts that promoted tumor development (15). In the model of colon cancer blocking EV-derived CD9 by antibody prevented the morphological transformation and migratory phenotype of cancer cells that uptake EVs (64). The CD9-positive EVs were higher in patients with prostate cancer compared to ones with benign prostate hyperplasia, and its secretion can be modulated in response to dihydrotestosterone. Importantly, siRNA knockdown of endogenous CD9 reduced cellular proliferation and expression of AR and prostate-specific antigen. However, knockdown of AR did not alter CD9 expression, implicating CD9 as an upstream regulator of AR (65). The exosomes may also determine organotropism and prepare the pre-metastatic niche in the sense of Stephen Paget´s seed and soil hypothesis (17, 66). Cancer cells from primary tumors release oncogenic biomolecules to the distant site before the cell invasion occurs, forming a pre-metastatic niche in the target organ that promotes successful metastatic outgrowth (16–20).

Therapy resistance in cancer can also occur *via* exosomes in several ways (17). Corcoran et al. reported the transfer of MDR1 by exosomes which enhanced a docetaxel efflux out of the recipient cells (67). Exosomes from drug-resistant breast cancer cells could also transmit chemoresistance do adriamycin and docetaxel by a horizontal transfer of microRNAs (68). Aung et al. have shown that tumor‐derived exosomes can protect cancer cells by transporting an abundance of proteins targeted by drugs, hence neutralising the therapy effects (23). Similarly, cells of the tumor microenvironment also release exosomes that can enhance drug resistance in cancer cells. For example, fibroblast‐derived exosomes have been shown to decrease the efficiency of chemotherapy and radiation in cancer by activating STAT1 and NOTCH3 signaling, which resulted in the expansion of therapy-resistant tumor-initiating cells (69). Chemotherapeutic drugs may also be excreted from cancer cells *via* exosomes (21, 22). Together, these studies described various mechanisms of exosome‐mediated drug resistance either through pumping anticancer drugs out of cells or transferring molecular cargo between cells.

## 4 CD9 in cancer cells: Dr. Jekyll and Mr. Hyde

CD9 expression is deregulated in a number of pathologies, including cancer, but the precise mechanism underlying these changes and the associated consequences are not fully understood (4). Bioinformatic analysis of binding sites in the promoter/enhancer region of the CD9 gene identified E2F, NFkB, SP1 and STAT3 as top transcription factors often associated with both the process of carcinogenesis and disease prognosis (70). Nevertheless, the CD9 protein plays a dual role in cancer progression, exhibiting both tumor-supportive and tumor-suppressive properties that are context-dependent.

### 4.1 Tumor-promoting properties

The plasma membrane protrusions enable the spreading of neoplastic cells, helping them to move between and invade surrounding stromal cells. In addition to passing through intercellular gap junctions, the neoplastic cell can also use a transcellular route for intra/extravasation, which are the essential steps of the metastatic process (71). Overexpression of CD9 has been shown to enhance FAK phosphorylation and reorganisation of the cortical actin cytoskeleton in fibrosarcoma HT1080 cells plated on laminin (5). This was also associated with the induction of MMP2 and PI3K-dependent signaling (6). Aggressive triple-negative breast cancer MDA-MB-231 cells displayed significant alterations of their plasma membrane protrusions after CD9 knockdown and had reduced tumorigenic and metastatic capacity in mouse xenografts (72). The potential role of CD9 in metastasis was indicated in a different study, describing increased expression of CD9 in breast cancer bone metastasis compared to primary tumors, where CD9 antibody treatment *in vivo* moderately inhibited the progression of bone lesions (73). In this sense, CD9 expression and migration were induced by native type IV collagen through a DDR1-dependent pathway in the breast cancer model, but not in non-tumorigenic MCF10A and MCF12A cells (66). Surprisingly, along with the plasma membrane, CD9 can also localize in nuclei and its depletion led to polynucleation and multipolar mitosis (74).

CD9 has also been shown to interact with VEGFR3 signaling. After intrathoracic implantation of lung cancer cells, metastasis to lymph nodes was diminished and accompanied by decreased neoangiogenesis and lymphangiogenesis in CD9 knock-out mice (12). Knocking down CD9 in human lymphatic endothelial cells also decreased their migration, proliferation and tube formation which was associated with attenuated VEGFR3 signaling. Importantly, active redistribution of endothelial CD9 was also observed during interactions between melanoma and endothelial cells in an intravasation assay (13). Anti-CD9 monoclonal antibodies specifically inhibited the transendothelial migration of melanoma cells. Association of CD9 with transendothelial invasion has also been observed by immunohistochemistry in cervical cancer as well as in melanomas (75, 76). Similarly, Hori et al. found CD9 expression at severe vessel invasion in gastric cancer (77). CD9 upregulation was also detected in ovarian carcinomas by expression profiling and immunohistochemistry (7). The CD9 upregulation associated with enhanced expression of TNF-alpha and NFkB signaling and treatment with CD9 blocking antibody ALB6 resulted in reduced tumor growth *in-vivo* (7). CD9 can also attenuate EGF signaling pathways in gastrointestinal cancer cells by colocalizing with EGFR (8).

One of the key drivers of tumor progression are cancer stem cells (CSCs) capable to self-differentiate, self-renew and fueling tumor growth. It has been reported that CD9 identifies a subpopulation of pancreatic cancer stem cells (CSCs) able to initiate and sustain pancreatic cancer growth as demonstrated in CD9 deficient organoid and mice models (78). Mechanistically, CD9 promoted the plasma membrane localisation of the glutamine transporter ASCT2, enhancing glutamine uptake in cancer cells. CD9 has been identified as a marker of CSCs also in the glioblastoma model, where its disruption led to decreased cell proliferation, invasion, and inhibition of tumor growth (79, 80). Decreased cell migration was also reported in highly metastatic hepatocellular carcinoma cells upon CD9 silencing (81) or in breast cancer cells using CD9-binding peptide (82). The same group successfully reduced melanoma lung metastasis after peptide binding to tetraspanin CD9. The CD9-binding peptide impeded tetraspanin web formation, cancer cell invasion and significantly reduced secretion and uptake of cancer cell exosomes (83). Antibody targeting of CD9 in pancreatic cancer disrupted CD9/ADAM interactions and led to decreased proliferation, migration and colony formation (84).

Regarding small-cell lung cancer (SCLC), CD9 was expressed preferentially in SCLC tumors and metastases from three of seven relapsed patients, whereas chemonaïve primary tumors from 16 patients were CD9 negative with only one exception (85). Mechanistically, CD9 was upregulated in chemoresistant cell lines, which adhered more tightly to fibronectin *via* β1 integrin, but they were less motile than the respective chemosensitive parental lines, implying a potential role of CD9 molecule in the cell adhesion-mediated drug resistance (85).

### 4.2 Tumor-inhibiting properties

Regarding breast cancer, Remsik et al. observed the downregulation of CD9 in cancer cells that underwent epithelial-mesenchymal transition (EMT) both *in vitro* and *in vivo* (11). High CD9 expression is associated with epithelial phenotype and favorable prognosis regarding recurrence-free survival (11). However, further mechanistic studies will be needed to clarify the role of CD9 in EMT and breast cancer progression. In ovarian cancer, the downregulation of CD9 attenuated the expression of several integrins and rearranged junctional and cytoskeletal molecules which was associated with weaker adhesion to the extracellular matrix (10). Enhanced peritoneal dissemination was observed for subclones with low CD9 expression (10), consistent with a previous report of inverse correlation of CD9 and ovarian cancer tumor stage (86). Decreased CD9 clustering may reflect the tendency of malignant cells to have less organized cell-cell junctions where tetraspanins are typically known to be clustered (3). Low affinity anti-CD9 antibody, C9BB, which binds preferentially to CD9 homodimer was used in experiments documenting a shift to heterodimers in cancer cells. This may be associated with decreased CD9 palmitoylation or altered expression of CD9 partners (3).

Takeda et al. observed a decreased lymph node metastasis of lung cancer cells transduced with CD9 without impact on the primary tumor growth (87). Similarly, ectopic expression of CD9 in fibrosarcoma cell line HT1080 reduced their lung metastatic ability by forming a complex with podoplanin, suppressing podoplanin-induced platelet aggregation (88). In line with these studies, genetic ablation of CD9 in a model of mouse prostate adenocarcinoma did not affect primary tumor development. Still, it increased the incidence of metastases to the liver but not the lungs, suggesting a possible tissue-specific manner of CD9 interactions in this model (89).

The antiproliferative effect of CD9 was also observed in *in vitro* model of human glioblastoma executed *via* inhibition of EGFR phosphorylation (9). In contrast, the downregulation of CD9 promoted cancer growth and metastasis through the upregulation of EGF in pancreatic cancer (90). In the context of SCLC that develops distant metastases extremely early, Funakoshi et al. observed the downregulation of tetraspanin CD9 in all cell lines. CD9 recovery suppressed cell motility of SCLC cells, suggesting that low expression of CD9 affects cell motility and may contribute to the highly invasive and metastatic phenotype of SCLC (91). Likewise CD9 overexpression in hepatocellular carcinoma inhibited proliferation *in vitro* and *in vivo* while CD9 knockdown enhanced *in vivo* growth (92).

## 5 Prognostic value of CD9 in solid tumors and a problem of different antibodies

Besides studies dealing with the molecular function of CD9, expression of this gene was also monitored in large cancer patient cohorts concerning tumor aggressiveness and survival. Relevant articles since 1993 are summarized in **Table 1** and some are briefly commented on below. High expression of CD9 is mostly associated with good patient outcomes, with a few exceptions. These might be attributed to special cancer subtypes and different antibodies used for CD9 staining.

**Table 1:** (A) Summary of immunohistochemical studies – high CD9 expression associates with good clinical outcome.

Authors	Tumor Type	Patients	Detection method	Antibody clone	Source	Therapy	Clinical outcome with high CD9
Miyake et al., 1995 (93)	breast ca	143	IHC, RT-PCR, western blotting	m31-15	in-house	S	less aggressive disease
Arihiro et al., 1998 (94)	breast ca	93	IHC, western blotting	n.s.	Dako	S	good prognosis, low risk of LN metastases
Houle et al., 2002 (86)	ovarian ca	40	IHC	m31-15	in-house, Dr. Miyake	S/AC	less aggressive disease
Sauer et al., 2003 (75)	cervical ca	44	IHC	72F6	Novocastra	S	good prognosis
Miyamoto et al., 2001 (95)	endometrial ca	71	IHC	TP82	Temecula, CA	S	good prognosis
Wang et al., 2007 (27)	prostatic ca	167	IHC	72F6	Novocastra	S	less aggressive disease
Mhawech et al., 2003 (96)	urothelial blader ca	320	IHC	n.s.	Novocastra	TURBT	good prognosis
Ai et al., 2007 (97)	urothelial blader ca	52	IHC	n.s.	Jingmei Biotech	TURBT	good prognosis
Buim et al., 2010 (98)	oral squamous ca	179	IHC, RT-PCR	n.s.	Neomarkers	S	good prognosis
Kusukawa et al., 2001 (99)	oral squamous ca	78	IHC, western blotting	clone 007	in-house	S	good prognosis
Uchida et al., 1999 (100)	esophageal squamous ca	108	IHC	m31-15	in-house	S	good prognosis
Zou et al., 2012 (101)	ca of gallblader	108	IHC	n.s.	Dako	S	good prognosis
Khushman et al., 2019 (102)	pancreatic ca	29	IHC	n.s.	Abcam	S	less aggressive disease
Kim et al., 2016 (103)	colorectal ca	305	IHC	EPR2949	Abcam	S/AC	good prognosis in left-side tumors only
Hashida et al. 2003 (104)	colorectal ca	146	IHC, RT-PCR	m31-15	in-house	S	good prognosis
Amatya et al., 2013 (105)	mesothelioma	112	IHC	72F6	Novus Biologicals	S/AC/RT	good prognosis
Si et Hersey 1993 (106)	melanoma	55	IHC	FMC56	in-house, Dr. Zola	S	less aggressive disease
Woegerbauer et al., 2010 (107)	Merkell cell ca	25	IHC, RT-PCR, western blotting	RDI-MCD9	Fitzgerald	S/AC/RT	good prognosis
Kohmo et al., 2010 (85)	small cell lung ca	24	IHC, RT-PCR, western blotting, flow cytometry	72F6	Novocastra	AC	good prognosis
Adachi et al., 1998 (108)	non small lung ca	172	IHC, RT-PCR	m31-15	in house	S	good prognosis
Higashiyama et al., 1995 (109)	adenoca of lung	132	IHC, RT-PCR	m31-15	in house	S	good prognosis

AC, adjuvant chemotherapy; ca, carcinoma; IHC, immunohistochemitry; LN, lymph node; NAC, neoadjuvant chemotherapy; n.s. not specified; RT, radiotherapy; S, surgery; TURBT; transuretral resection of bladder tumor.

Discordant findings have been reported for breast, ovarian, melanoma, pancreatic and esophageal cancer. The good prognostic value of high CD9 expression was described in studies using the in-house mouse monoclonal antibody m31-15 (93, 100) and a monoclonal antibody from Dako (94). Of note, studies using the antibody clone m31-15 consistently report the good prognostic value of high CD9 in all cancer types (see **Tables 1A**, **1B**). On the other hand, other groups reported poor outcomes for breast and ovarian cancer patients with high CD9 expression using Abcam monoclonal antibody EPR2949 or an antibody from Millipore (7, 113, 114). Kwon et al. also evaluated stromal immune cells and their CD9 expression was associated with good patient outcome (113). The same group used the EPR2949 antibody also for colorectal cancer where the high CD9 expression in tumors was associated with a good prognosis (103). Regarding stroma, high stromal CD9 evaluated with antibody clone C-4 was also associated with better survival of patients with pancreatic ductal adenocarcinoma, while positive tumor CD9 showed opposite results (115).

**Table 1:** (B) Summary of immunohistochemical studies – high CD9 expression associates with poor clinical outcome.

Authors	Tumor Type	Patients	Detection method	Antibody clone	Source	Therapy	Clinical outcome with high CD9
Kim et al., 2019 (110)	papillary thyroid ca	553	IHC	n.s.	Novocastra	S	associated with LN metastases
Hori et al., 2004 (77)	gastric ca	78	IHC, nothern and western blotting	72F6 and 007	Novocastra and Dr. Mekada, resp.	S/AC	associated with advanced disease
Soyuer et al., 2010 (111)	gastric ca	49	IHC	72F6	Novocastra	S/AC	poor prognosis
Miki et al., 2018 (14)	gastric ca	619	IHC	n.s.	Life Technologies	S	poor prognosis, in particular of the scirrhous type
Huan et al., 2015 (112)	esophageal squamous ca	104	IHC, western blotting	n.s.	Santa Cruz	S	poor prognosis, associated with advanced disease
Kwon et al., 2017 (113)	breast ca	1349	IHC	EPR2949	Abcam	S/AC	poor prognosis in luminal A subtype
Baek et al., 2019 (114)	lobular breast ca	113	IHC	EPR2949	Abcam	S/AC/RT	poor prognosis
Hwang et al.2012 (7)	ovarian ca	30	IHC, microarray, RT-PCR, western blotting	n.s.	Millipore	S	associated with advanced disease
Lucarini et al., 2022 (76)	cutaneous melanoma	120	IHC	72F6	Novocastra	S	poor prognosis, associated with LN metastases
Han et al., 2022 (115)	pancreatic ca, NAC	179	IHC	C-4	Santa Cruz	NAC/S	poor prognosis

AC, adjuvant chemotherapy; ca, carcinoma; IHC, immunohistochemitry; LN, lymph node; NAC, neoadjuvant chemotherapy; n.s. not specified; RT, radiotherapy; S, surgery; TURBT; transuretral resection of bladder tumor.

Another frequently used antibody is clone 72F6 from Novocastra. High levels of CD9 evaluated with this antibody were associated with poor prognosis in gastric cancer (111), while the association with good prognosis was found for mesothelioma, cervical and prostate cancer (75, 105, 116). Several other studies used an antibody from Novocastra without closer specification, complicating their findings’ interpretation. One of these studies investigated breast cancer samples and found no prognostic value of CD9 (117).

Overall, the interpretation of immunohistochemistry studies is problematic due to the use of different antibodies that are not thoroughly validated and essential details are missing. For example, epitopes for the most frequently used monoclonal antibodies m31-15, 72F6 and EPR2949 are unavailable. Two recent meta-analyses concluded that low CD9 expression is significantly associated with poor prognosis of cancer patients (118, 119) however, these results are oversimplified and problematic with respect to the abovementioned issues.

As described in the previous chapter, CD9 is not clearly associated with either tumor suppression or promotion. Additional mechanistic studies have recently been thoroughly reviewed by Lorico et al. They also summarized CD9 targeting with therapeutic antibodies and drew attention to the potential side effects of this strategy. This approach might further be complicated by the presence of CD9 in exosomes which may neutralize the therapeutic antibodies or enhance the pro-metastatic effects of exosomes by their enhanced endocytosis (53).

## 6 Conclusion

A growing body of evidence points to the important role of CD9 in various physiological processes and cancer. As highlighted in the review, CD9 is not unequivocally associated with either tumor suppression or promotion, and the antibodies used to detect CD9 might be problematic. Despite conflicting results in different types of cancer, the clinical relevance of CD9 has been highlighted by several immunohistochemical and, more importantly, mechanistic studies. Therapeutic targeting of CD9 is emerging, however, this approach may be complicated by the presence of CD9 in exosomes, which may neutralize therapeutic antibodies or enhance the pro-metastatic effects of exosomes by their increased endocytosis. In conclusion, fully validated antibodies and well-designed functional studies may help to elucidate further the role of CD9 in cancer progression and patient clinical outcome.

## Author contributions

JB, SB, KS – conceptualization. RO – writing, preparation of original draft. BK, KK, KS – writing, reviewing and editing. KK - graphical figures preparation. JB - supervision, writing, reviewing and editing. All authors contributed to the article and approved the submitted version.
